# The effect of nephrectomy on Klotho, FGF-23 and bone metabolism

**DOI:** 10.1007/s11255-017-1519-9

**Published:** 2017-01-27

**Authors:** Katarzyna Kakareko, Alicja Rydzewska-Rosolowska, Szymon Brzosko, Joanna Gozdzikiewicz-Lapinska, Ewa Koc-Zorawska, Pawel Samocik, Robert Kozlowski, Michal Mysliwiec, Beata Naumnik, Tomasz Hryszko

**Affiliations:** 1 I Department of Nephrology and Transplantation with Dialysis Unit, Medical University of Bialystok, Ul. Zurawia 14, 15-540 Białystok, Poland; 2 II Department of Nephrology and Hypertension with Dialysis Unit, Medical University of Bialystok, Białystok, Poland; 3 Department of Oncological and General Urology, J. Sniadecki Provincial Hospital, Białystok, Poland

**Keywords:** Acute GFR decline, Nephrectomy, Klotho, FGF-23, Bone metabolism

## Abstract

**Background:**

Increased concentration of fibroblast growth factor 23 (FGF-23) and decreased levels of soluble Klotho (sKL) are linked to negative clinical outcomes among patients with chronic kidney disease and acute kidney injury. Therefore, it is reasonable to hypothesize that GFR reduction caused by nephrectomy might alter mineral metabolism and induces adverse consequences. Whether nephrectomy due to urological indications causes derangements in FGF-23 and sKL has not been studied. The aim of the study was to evaluate the effect of acute GFR decline due to unilateral nephrectomy on bone metabolism, FGF-23 and sKL levels.

**Methods:**

This is a prospective, single-centre observational study of patients undergoing nephrectomy due to urological indications. Levels of C-terminal FGF-23 (c-FGF-23), sKL and bone turnover markers [β-crosslaps (CTX), bone-specific alkaline phosphatase (bALP) and tartrate-resistant acid phosphatase 5b (TRAP 5b)] were measured before and after surgery (5 ± 2 days).

**Results:**

Twenty-nine patients were studied (14 females, age 63.0 ± 11.6, eGFR 87.3 ± 19.2 ml/min/1.73 m^2^). After surgery, eGFR significantly declined (*p* < 0.0001). Nephrectomy significantly decreased sKL level [709.8 (599.9–831.2) vs. 583.0 (411.7–752.6) pg/ml, *p* < 0.001] and did not change c-FGF-23 concentration [70.5 (49.8–103.3) vs. 77.1 (60.5–109.1) RU/ml, *p* = 0.9]. Simultaneously, alterations in bone turnover markers were observed. Serum concentration of CTX increased [0.49 (0.4–0.64) vs. 0.59 (0.46–0.85) ng/ml, *p* = 0.001], while bALP and TRAP 5b decreased [23.6 (18.8–31.4) vs. 17.9 (15.0–22.0) U/l, *p* < 0.0001 and 3.3 (3.0–3.7) vs. 2.8 (2.3–3.2) U/l, *p* < 0.001, respectively].

**Conclusions:**

Nephrectomy among patients with preserved renal function before surgery does not increase c-FGF-23 but reduces sKL. Moreover, nephrectomy results in derangements in bone turnover markers in short-term follow-up. These changes may participate in pathogenesis of bone disease after nephrectomy.

## Introduction

Fibroblast growth factor 23 (FGF-23) and Klotho are key players in maintaining mineral homoeostasis. Increase in FGF-23 concentration is accompanied by Klotho reduction as chronic kidney disease (CKD) worsens [1, 2]. Both molecules have gained attention for their association with important clinical outcomes such as cardiovascular incidents [3] and mortality [4]. It has been thought that FGF-23 and Klotho may link bone mineral disturbances with cardiovascular mortality.

Recently, it was shown that among patients with acute kidney injury (AKI), FGF-23 concentrations are elevated and associated with negative clinical outcomes [5, 6]. In contrast, there is little data on FGF-23 and soluble Klotho (sKL) after mild-to-moderate reduction in glomerular filtration rate (GFR), which is evoked by nephrectomy. This might be especially important with regard to living kidney donation and patients undergoing nephrectomy due to urological indications in light of negative connotations between FGF-23 and clinical consequences.

It is a matter of debate whether and how acute GFR decline predisposes to bone metabolism disturbances. The impact of those changes on bone metabolism has been studied in two groups of patients: living kidney donors and patients undergoing nephrectomy. On the one hand, nephrectomy due to renal cell carcinoma is a significant risk for osteoporosis and increased fracture risk [7], but, on the other hand, data concerning living kidney donors are conflicting. It is reported that even though living kidney donors experience alterations of hormones involved in bone metabolism [8–10], there is no increased fracture risk [11]. The precise mechanism by which acute GFR decline predisposes to bone metabolism disturbances is largely unknown.

A nephrectomy due to urological indications in patients with preserved renal function offers unique clinical model to study effect of acute mild-to-moderate GFR decline, as the patients serve as their own controls without any confounding factors always present among patients with CKD and even more pronounced in AKI setting.

Regarding the fact that alterations in FGF-23 and sKL disturb bone health early in the course of CKD, the study was undertaken to evaluate the effect of acute GFR decline due to unilateral nephrectomy on bone metabolism, FGF-23 and sKL levels. The secondary aim was to assess whether changes in the concentration of the above molecules evoked by nephrectomy are associated with alterations in the markers of bone formation and/or resorption.

## Materials and methods

### Study design

This study is a post hoc analysis of frozen blood and urine samples from the previously reported data [12]. The former study was designed as a prospective, single-centre trial and patient served as a self-control group. All patients undergoing nephrectomy due to urological indications in the Department of Oncological and General Urology, Sniadecki Provincial Hospital in Bialystok (Poland), were enrolled unless end-stage renal disease requiring dialysis (four patients) or lack of informed consent (five patients) was present.

After the surgery, all the patients underwent standard hydration protocol with isotonic saline. During the first 24 h, 2500 ml of intravenous fluid was administered and nil per os was prescribed; on the second day, 1000 ml of intravenous fluid was given and patients were allowed to start progressive fluid oral intake. From the third day, no intravenous fluids were given.

The study was approved by the local ethics committee. The protocol adhered to the principles of the Declaration of Helsinki and written informed consent was obtained from each participant.

### Laboratory measurements

Urine and venous fasting blood samples were collected on the morning prior to surgery and after nephrectomy (5 ± 2 days after nephrectomy depending on the duration of hospitalization). To prevent possible changes due to intra-day variability, our samples were collected always at early morning, after overnight fasting. Plasma, serum and urine were centrifuged, aliquoted and frozen at −70 °C until assayed.

Serum soluble Klotho and plasma C-terminal FGF-23 (c-FGF-23) levels were measured using the enzyme-linked immunosorbent assay (ELISA) kit (IBL, International GmbH, Hamburg, Germany; intra-assay CV < 4% and Immutopics, Inc., San Clemente, CA, USA; intra-assay CV < 2.5%, respectively). Serum bone-specific alkaline phosphatase (bALP) was determined by an ELISA kit from MicroVue (Quidel Corporation, San Diego, CA, USA; intra-assay CV < 6%). Tartrate-resistant acid phosphatase 5b (TRAP 5b) was measured in serum by an ELISA kit from MicroVue (Quidel Corporation, San Diego, CA, USA; intra-assay CV < 2.2%). ELISA immunoassay was used to measure serum cross-linked C-telopeptide of type 1 collagen (CTX, CrossLaps, IDS Nordic A/S, Herlev, Denmark; intra-assay CV < 6%). Plasma intact PTH levels were measured using Immutopics assay (Inc., San Clemente, CA, USA). All samples were assayed according to manufacturer’s recommendations. For sKL, samples were diluted two times. Blood and urine levels of creatinine, calcium and phosphate were determined with standard laboratory methods.

### Calculations and statistics

To estimate phosphate and calcium renal handling, we used the ratio of tubular maximum reabsorption to the glomerular filtration rate (TmPO_4_/eGFR—as an index for the phosphate renal threshold) and urinary fractional excretion of calcium (FE_Ca_). The calculations were made as follows:

- TmPO_4_/eGFR = PO_4_ * (1 − Fe_PO4_) if Fe_PO4_ ≥ 0.2 otherwise TmPO_4_/eGFR = PO_4_ * e (10.318 * Fe_PO4_^2^ − 5.18 * Fe_PO4_ + 0.4), where urinary fractional excretion of phosphate (Fe_PO4_) was computed as below:
- Fe_PO4_ = [(urinary phosphate × serum creatinine)/(serum phosphate × urinary creatinine)] [13, 14],
- and FE_Ca_ = [(urinary calcium × serum creatinine)/(serum calcium × urinary creatinine)] × 100%.

GFR was estimated using CKD epidemiology collaboration (CKD-EPI) equation [15].

Variable distribution was tested with the Shapiro–Wilk W test of normality. The normally distributed data were presented as mean ± 1 SD, the skewed data as median (interquartile range; IQR). Before statistical computations, logarithmic transformations were performed on skewed variables to obtain normal distribution, if possible. Student’s *t* test for paired samples or Wilcoxon signed-rank test was used to compare continuous variables at selected time points. Changes in measured parameters were expressed as delta (∆) and calculated as follows: postoperative minus preoperative value. Associations between deltas were assessed using bivariate correlations with Pearson or Spearman’s test depending on meeting the assumptions. Mixed regression analyses accounting for time effect were performed in search for longitudinal associations between variables of interest. Results were reported as beta coefficient *β* and 95% confidence intervals (95% CI). A two-tailed *p* value of <0.05 was considered statistically significant. All computations were performed with Statistica 12 (StatSoft, Tulsa, OK, USA).

## Results

### Characteristics of the study population

We enrolled twenty-nine patients (14 females) who underwent nephrectomy. The mean baseline eGFR was 87.3 ± 19.2 ml/min/1.73 m^2^. Additional baseline characteristics of the studied population are detailed in Table 1. After nephrectomy in four subjects anuria occurred, thus parameters evaluated in urine were measured in 25 subjects. In these patients, hydration protocol was violated, as they received fluids according to their condition. Statistical computations were repeated after exclusion of these cases and yielded similar results. None of the patients required dialysis during the study duration.

**Table 1:** Baseline characteristics of the study population

Characteristic	Value
Age (years)	63.0 ± 11.6
Sex	
Male	15 (52%)
Female	14 (48%)
Nephrectomy	
Partial	8 (28%)
Radical	21 (72%)
Indication for nephrectomy	
Tumour	21 (72%)
Cirrhotic kidney	4 (14%)
Complex renal cysts	2 (7%)
Kidney stones	1 (3.5%)
Pyonephrosis	1 (3.5%)
eGFR, ml/min/1.73 m^2^	87.3 ± 19.2

Data are mean ± SD or number (percentage)

Groups with partial and radical nephrectomy and with tumour and non-tumour did not differ significantly concerning evaluated parameters except from non-significant difference in baseline value of sKL between tumour and non-tumour group (tumour group—680.2 ± 131.2 vs. non-tumour group—805.3 ± 185.9, *p* = 0.05).

### Effect of nephrectomy on calcium, phosphate and iPTH

Biochemical parameters before and after nephrectomy are presented in Table 2. As expected, eGFR declined significantly after surgery compared with the baseline values (87.3 ± 19.2 vs. 69.8 ± 24.7 ml/min/1.73 m^2^, *p* < 0.0001). We observed significant decline in serum calcium and phosphate concentration (*p* < 0.0001 and *p* = 0.002, respectively), while urinary calcium (*p* = 0.02) and phosphate excretion (reduction in TmPO_4_/eGFR, *p* = 0.001) increased. There was no significant change in intact PTH concentration during the study period.

**Table 2:** Changes in biochemical parameters after nephrectomy

Parameter	Baseline	After nephrectomy	*p* value
eGFR (ml/min/1.73 m^2^)	87.3 ± 19.2	69.8 ± 24.7	*p* < 0.001
Creatinine (mg/dl)^a^	0.82 (0.56–1.7)	1.02 (0.58–6.28)	*p* < 0.001
Calcium (mmol/l)	2.31 ± 0.11	2.17 ± 0.15	*p* < 0.001
Phosphate (mmol/l)	1.14 ± 0.2	0.97 ± 0.24	*p* = 0.002
iPTH (pmol/l)	3.12 (2.02–3.66)	2.68 (2.04–4.51)	*p* = 0.6
Soluble Klotho (pg/ml)	709.8 (599.9–831.2)	583.0 (411.7–752.6)	*p* < 0.001
c–FGF-23 (RU/ml)	70.5 (49.8–103.3)	77.1 (60.5–109.1)	*p* = 0.9
CTX (ng/ml)	0.49 (0.4–0.64)	0.59 (0.46–0.85)	*p* = 0.001
bALP (U/l)	23.6 (18.8–31.4)	17.9 (15.0–22.0)	*p* < 0.001
TRAP 5b (U/l)	3.3 (3.0–3.7)	2.8 (2.3–3.2)	*p* < 0.001
FE_Ca_ (%)	0.67 (0.33–0.85)	1.14 (0.59–1.38)	*p* = 0.02
TmPO_4_/eGFR (mmol/l)	1.07 (0.84–1.35)	0.8 (0.64–0.93)	*p* = 0.001

Data are presented as number (percentage), mean ± SD or median (interquartile range)

*eGFR* estimated glomerular filtration rate, *iPTH* intact parathyroid hormone, *c-FGF-23* c-terminal fibroblast growth factor 23, *CTX* cross-linked C-telopeptide of type 1 collagen, *bALP* bone-specific alkaline phosphatase, *TRAP 5b* tartrate-resistant acid phosphatase 5b, *FE*_*Ca*_ urinary fractional excretion of calcium, *TmPO*_*4*_ */eGFR* ratio of tubular maximum reabsorption rate to the glomerular filtration rate

^a^For creatinine, data are presented as the median (min–max)

Phosphatemia changes [ΔP = −0.54 (−1.01 to 0.11) mmol/l] were positively associated with ΔiPTH [∆ = 0.21 (−0.44 to 0.92) pmol/l; *r* = 0.6, *p* = 0.002] and ∆c-FGF-23 [∆ = 3.0 (−15.49 to 26.29) RU/ml; *r* = 0.6, *p* = 0.0001]. Phosphate level was determined by c-FGF-23 [*β* = 1.68 (95% CI 0.82–2.53), *p* < 0.001], iPTH [*β* = 0.41 (95% CI 0.08–0.74), *p* = 0.016] in mixed regression analysis model after adjustment for time effect and kidney function.

Alterations in serum calcium evoked by nephrectomy (ΔCa = −0.15 ± 0.15 mmol/l) were associated negatively with iPTH alterations (ΔiPTH) (*r* = −0.4, *p* = 0.03) but not with any evaluated parameters. Serum calcium level was associated only with iPTH [*β* = −0.44 (95% CI −0.78 to (−0.10)), *p* = 0.013] in mixed model adjusted for time effect and renal function.

### Effect of nephrectomy on c-FGF-23, sKL

**Fig. 1:**
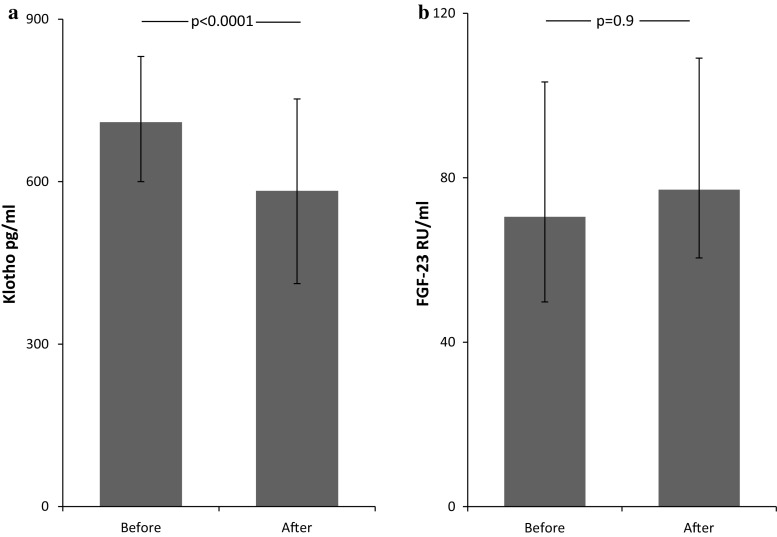
Impact of nephrectomy on serum levels of **a** soluble Klotho, **b** fibroblast growth factor (FGF-23). Data are presented as median and interquartile range

There was a significant decrease in sKL level (*p* < 0.0001) after nephrectomy (Fig. 1a). Serum concentration of c-FGF-23 was not changed by the procedure (Fig. 1b).

Alterations of c-FGF-23 concentration (Δc-FGF-23) were associated with ΔP (*r* = 0.6, *p* = 0.0001) and ΔeGFR (∆ = −17.45 ± 19.62 ml/ml/1.73 m^2^; *r* = −0.4, *p* = 0.04).

Mixed regression analyses adjusted for time effect, renal function, and iPTH showed phosphate [*β* = 0.24 (95% CI 0.12–0.36), *p* < 0.001] to be determinant of c-FGF-23 level.

Change in sKL [ΔsKL = −148.6 (−237.1 to −56.8) pg/ml] was negatively associated with ΔCTX [∆ = 0.18 (0.01–0.27) ng/ml; rho = −0.4, *p* = 0.03] and ΔiPTH (*r* = −0.6, *p* = 0.001).

Level of sKL remained significantly associated with iPTH (*β* = −0.47 [95% CI −0.70 to (−0.23)], *p* < 0.001) and CTX [*β* = −0.39 (95% CI −0.68 to (−0.09)), *p* = 0.013] after adjustment for renal function in mixed regression analysis.

### Effect of nephrectomy on markers of bone resorption and formation

**Fig. 2:**
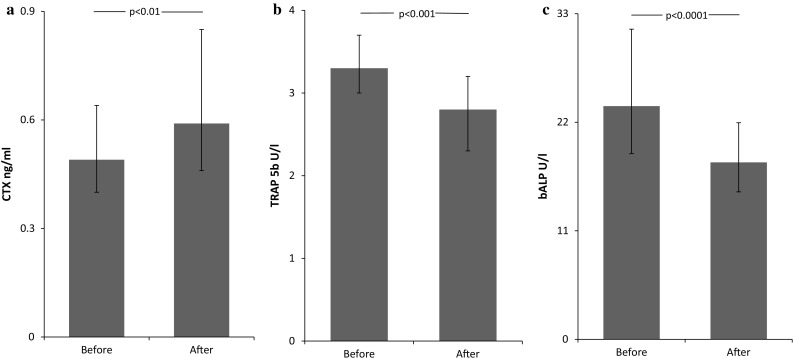
Impact of nephrectomy on serum levels of **a** cross-linked C-telopeptide of type 1 collagen (CTX), **b** tartrate-resistant acid phosphatase 5b (TRAP 5b) and **c** bone-specific alkaline phosphatase (bALP). Data are presented as median and interquartile range

Nephrectomy resulted in alterations of bone turnover markers. Serum CTX increased after surgery compared to baseline (*p* < 0.01; Fig. 2a), while serum TRAP 5b and bALP concentration decreased (*p* < 0.001; Fig. 2b and *p* < 0.0001; Fig. 2c).

Neither ΔCTX, ∆TRAP 5b nor ΔbALP correlated with ΔeGFR. ∆CTX evoked by nephrectomy was negatively associated only with changes in sKL concentration (rho = −0.4, *p* = 0.03) and with alterations of serum phosphate (rho = 0.4, *p* = 0.04). In mixed regression model accounting for time effect, none of aforementioned parameters significantly predicted CTX concentration.

Changes of any evaluated parameters were not associated with bALP (ΔbALP) and TRAP 5b (∆TRAP 5b) alterations.

## Discussion

The aim of this study was to test the hypothesis, whether nephrectomy changes the concentration of c-FGF-23, sKL and affects bone metabolism. Our data show that acute reduction in GFR after nephrectomy does not alter c-FGF-23 concentration in short-term follow-up. It is in line with recent study performed in living kidney donors, which showed rather FGF-23 reduction but not the increase in short term [8]. On the other hand, we are aware that decrease in calcium concentration, observed in our study, might prevent FGF-23 increase, as was shown previously [16]. However, we did not observe any association between calcium and FGF-23 changes. These results are in contrast to data obtained from patients with AKI, in whom elevated FGF-23 levels are observed already after 1-h of AKI onset, with more than tenfold rise at 24 h [5]. Moreover, in human study it was shown that FGF-23 increase, at the end of cardiac surgery predicted AKI development [6]. The mechanisms underlying augmented FGF-23 levels in patients with AKI are unknown. Our data shed some light on potential mechanism, as it shows there are two possible explanations. Either there must be a threshold of acute GFR decline beyond which FGF-23 production escalates or there is a different factor involved, associated with acute state in the setting of AKI, activating FGF-23 increase.

Currently two assays measuring FGF-23 levels are available. Intact FGF-23 (iFGF-23) assay that detect only a biologically active form and second one, used in our study, an assay measuring both c-terminal fragments and intact form of FGF-23. Thus, if intact form of FGF-23 is elevated then the c-terminal assay also would detect the rise. Of course when c-terminal assay detects elevation in FGF-23 it is difficult to say if there is a rise in iFGF-23 or increase in FGF-23 cleavage. Since we did not observe any changes in c-terminal FGF-23 assay, we can hypothesize that in our study neither no increased synthesis, nor degradation occurred. However, without c-FGF-23/iFGF-23 ratio to support our opinion, an alternative explanation is possible. Theoretically, no change in c-terminal FGF-23 measurement could be due to decrease in intact and an increase in c-terminal FGF-23 fragments. Although already published data where both forms of FGF-23 were measured during AKI [5, 6, 17], universally showed increase in c-FGF23 as well as iFGF-23. Thus, we believe that our data add interesting point to the discussion regarding mutual relations between acute GFR decrease and FGF-23 behaviour in different clinical settings. Even though our sample size was small, it provided approximately 80% power to detect at least 11% change in c-FGF-23 concentration at 5% significance level, based on power calculations based on two previously published data (for iFGF-23 after nephrectomy [18], and c-FGF-23 after hip replacement procedure [19]).

Whether nephrectomy induces FGF-23 increase with all its negative consequences is especially important for living kidney donors. Data from kidney donors showed that in long-term follow-up after nephrectomy, FGF-23 increases [9, 10, 18]. These results differ from those of our study, although we investigated short-term changes and our study population were patients with urological indications for nephrectomy. We may hypothesize that the increase in FGF-23 occurs at higher GFR decrease than observed in our study or our follow-up was relatively too short to detect changes. A rise in FGF-23 is an effect of GFR decline, and time is essential for those changes to become evident.

Our finding that FGF-23 levels are constant, despite nephrectomy is especially interesting in the context of results obtained by Goebel et al., who reported significant increase in FGF-23 after orthopaedic procedures [19]. It points that rather other stimuli than surgery by itself induces this molecule’s production. This hypothesis is in line with data obtained in animal experiment where sham-operated rats FGF-23 levels were stable [20]. Results from ICU patients, where FGF-23 increase was observed despite normal renal function, make the question about factors determining FGF-23 levels even more intriguing [21].

In agreement with previous reports [22], we observed a significant decrease in serum sKL after renal mass reduction, reaffirming the statement that kidneys are the major source of sKL. Study in living kidney donors with longer follow-up demonstrated that sKL increased after the initial postoperative reduction but still was lower than pre-donation [8]. Apart from Klotho’s role in ageing and phosphate homoeostasis [23], its soluble form acts as an endocrine protein that exerts pleiotropic actions, including protection of endothelial function by its antioxidant properties, inhibition of vascular calcification, suppression of fibrosis and inflammation [24, 25]. Reduced sKL may therefore contribute to many complications but how it translates to any patient-oriented measures is still a matter of debate. Even though sKL is discussed as a novel biomarker for progression in CKD [26], there are conflicting data from human studies regarding influence of sKL levels on clinical outcomes, reporting both positive [27] as well as neutral effects [28]. Acute reduction in GFR resulted in derangements in minerals handled by the kidneys, e.g., decreased serum calcium and phosphate levels. The most probable cause of reduction in phosphate concentration was increase in renal losses. Our data showed also increased calcium fractional excretion after nephrectomy. Even though decreased serum calcium is connected to disturbed vitamin D metabolism [29], hypocalcemia may not be specific only to nephrectomy, since hypocalcemia and secondary increase in PTH were observed after different abdominal surgeries [30]. In our opinion, hypercalciuria may be partly caused by sKL reduction, as lack of this molecule diminishes the activity of TRPV5 channels [31] and leads to increased renal calcium excretion although we did not observe significant association between the degree of calciuria and magnitude of sKL reduction.

Another compelling finding of the present study is that an acute decline in GFR, caused by nephrectomy, is associated with the alterations in both markers of bone metabolism: resorption and formation. We have shown in our study the rise in CTX and decrease in bALP and TRAP 5b after nephrectomy. This finding seems to be conflicting as CTX and TRAP 5b are both markers of resorption, and we have found their opposite behaviour after surgery. Since CTX tends to accumulate as the renal function declines [32, 33], TRAP 5b, which is degraded in the liver, is more accurate in our study population, because kidney function has no effect on circulating TRAP 5b activity [34, 35].

Our study suggests that surgically induced nephron loss leads to decrease in bone turnover, which could disintegrate bone homoeostasis. Although we are aware that due to short-term evaluation in our study, one may speculate that reported changes are rather the effects of the surgery but not renal mass reduction. However, after hip replacement surgery, one week after procedure, no changes in serum calcium, phosphorus, total alkaline phosphatase and urinary phosphorus were reported [36]. Based on current sparse data from human [36] and animal studies [20], it seems that rather nephrectomy but not the surgery induces changes in bone metabolism. Broader impact of the changes initiated by nephrectomy is unclear. Animal work shows that subtotally nephrectomized rats had alterations of the structural and mechanical properties of cortical bone material [37]. Human data reported by Bagrodia et al. [7] revealed the association of radical nephrectomy with higher risk of postoperative osteoporosis and fractures, showing the superiority of partial over radical nephrectomy. On the other hand, in the study of kidney donors, the fracture rate was not significantly higher compared to controls [11], although others reported disturbances in bone metabolism markers [10]. Further studies concerning this matter are needed. If the association between nephrectomy and disturbed bone metabolism is confirmed in a larger study, prevention of changes in bone health might become important concern in management of kidney donors and urological patients.

There are some limitations of this study. We are aware that there is a difficulty in assessment parameters changes after nephrectomy, caused by the possible influence of the fasting before operation, undergoing anaesthesia and intravenous hydration. However, at least with regard to c-FGF-23, recently published study showed that acute volume changes do not impact its measurements [38]. The study population consisted of patients with various indications for nephrectomy; therefore, we cannot exclude that the presence of different diseases had an impact on our conclusions. Since our results were uniform and each patient served as their own control, it seems very unlikely although cannot be ruled out. Moreover, our aim was to evaluate the impact of the nephrectomy on FGF-23 and sKL concentrations in patients with preserved renal function; nevertheless, we did not study living kidney donors. Thus, our study’s findings might not be relevant to this specific group. Additional study with longer follow-up is needed to confirm that our results are consistent over the time. However, we think that this study makes interesting point in the discussion about FGF-23 short-term behaviour after kidney injury, showing that nephrectomy may differ from other types of kidney damage.

The study was not designed to examine patients’ outcomes, which does not allow for conclusions regarding causality. Finally, the comparator group and assessing the degree of bone loss by a bone mineral density technique would undoubtedly strengthen our results.

In summary, we present data showing neutral effect of GFR reduction on FGF-23 concentration of patients undergoing nephrectomy due to urological indications. Evoked reduction in renal mass causes decrease in sKL level. Whether it translates to patient-oriented clinical outcomes requires further investigation. Moreover, nephrectomy resulted in derangements in bone turnover markers. These changes may participate in pathogenesis of bone disease after nephrectomy.
